# SARS-CoV-2-Mediated Lung Edema and Replication Are Diminished by Cystic Fibrosis Transmembrane Conductance Regulator Modulators

**DOI:** 10.1128/mbio.03136-22

**Published:** 2023-01-10

**Authors:** Jose M. Honrubia, Javier Gutierrez-Álvarez, Alejandro Sanz-Bravo, Ezequiel González-Miranda, Diego Muñoz-Santos, Carlos Castaño-Rodriguez, Li Wang, Marta Villarejo-Torres, Jorge Ripoll-Gómez, Ana Esteban, Raul Fernandez-Delgado, Pedro José Sánchez-Cordón, Juan Carlos Oliveros, Stanley Perlman, Paul B. McCray, Isabel Sola, Luis Enjuanes

**Affiliations:** a Department of Molecular and Cell Biology, Centro Nacional de Biotecnología (CNB-CSIC), Madrid, Spain; b Department of Infectious Diseases and Global Health, Animal Health Research Center (CISA), National Institute of Research, Agricultural and Food Technology (INIA-CSIC), Valdeolmos, Madrid, Spain; c Veterinary Pathology Department, Animal Health Research Center (CISA), National Institute of Research, Agricultural and Food Technology (INIA-CSIC), Valdeolmos, Madrid, Spain; d Bioinformatics for Genomics and Proteomics Unit, CNB-CSIC, Campus Universidad Autónoma de Madrid, Madrid, Spain; e Department of Microbiology, University of Iowa, Iowa City, USA; f Stead Family Department of Pediatrics, The University of Iowa, Iowa City, Iowa, USA; g Pappajohn Biomedical Institute, The University of Iowa, Iowa City, Iowa, USA; h Center for Gene Therapy, The University of Iowa, Iowa City, Iowa, USA; i Interdisciplinary Program in Immunology, University of Iowa, Iowa City, USA; NIAID, NIH

**Keywords:** antivirals, CFTR, coronavirus, envelope (E) protein, lung edema resolution

## Abstract

Coronaviruses (CoVs) of genera α, β, γ, and δ encode proteins that have a PDZ-binding motif (PBM) consisting of the last four residues of the envelope (E) protein (PBM core). PBMs may bind over 400 cellular proteins containing PDZ domains (an acronym formed by the combination of the first letter of the names of the three first proteins where this domain was identified), making them relevant for the control of cell function. Three highly pathogenic human CoVs have been identified to date: severe acute respiratory syndrome coronavirus (SARS-CoV) and Middle East respiratory syndrome coronavirus (MERS-CoV), and SARS-CoV-2. The PBMs of the three CoVs were virulence factors. SARS-CoV mutants in which the E protein PBM core was replaced by the E protein PBM core from virulent or attenuated CoVs were constructed. These mutants showed a gradient of virulence, depending on whether the alternative PBM core introduced was derived from a virulent or an attenuated CoV. Gene expression patterns in the lungs of mice infected with SARS-CoVs encoding each of the different PBMs were analyzed by RNA sequencing of infected lung tissues. E protein PBM of SARS-CoV and SARS-CoV-2 dysregulated gene expression related to ion transport and cell homeostasis. Decreased expression of cystic fibrosis transmembrane conductance regulator (CFTR) mRNA, essential for alveolar edema resolution, was shown. Reduced CFTR mRNA levels were associated with edema accumulation in the alveoli of mice infected with SARS-CoV and SARS-CoV-2. Compounds that increased CFTR expression and activity, significantly reduced SARS-CoV-2 growth in cultured cells and protected against mouse infection, suggesting that E protein virulence is mediated by a decreased CFTR expression.

## INTRODUCTION

Coronaviruses (CoVs) are cytoplasmic, positive-sense RNA viruses with a large genome (around 30 kb (kb)) (1). CoVs infect a wide variety of animals including humans. Seven human CoVs (hCoVs) have been identified. Four (229E, OC43, HKU1, and NL63) are responsible for approximately 15 to 30% of common colds (2, 3). In contrast, the other three zoonotic CoVs severe acute respiratory syndrome coronavirus (SARS-CoV), Middle East respiratory syndrome CoV (MERS-CoV), and SARS-CoV-2 are highly pathogenic.

SARS-CoV was first identified in China in 2002 and spread worldwide to infect more than 8,000 people with a mortality rate of around 10% (4, 5). In 2012, MERS-CoV was identified in Saudi Arabia (6, 7). Since then, 2,562 laboratory-confirmed cases and 890 deaths have been reported by WHO as of May 2022, corresponding to a fatality rate of 35% (https://www.who.int/emergencies/mers-cov/en/). SARS-CoV-2 was discovered in December 2019 and is the causative agent of COVID-19, the most important pandemic of the 21st century (8). Since then, the European Center for Disease Control (ECDC) has reported 600 million cases worldwide and more than 6,000,000 deaths with a mortality rate of 1.2% (https://www.ecdc.europa.eu/en/geographical-distribution-2019-ncov-cases). Studies of CoV-host interactions are essential to developing safe and effective antiviral therapies and vaccines, which are required to resolve the ongoing international health emergency and to prevent and treat future pandemics caused by emerging zoonotic highly pathogenic CoVs (9–11).

Our group has described that the envelope (E) protein of SARS-CoV and MERS-CoV is implicated in virus replication and virulence (12–14). Two domains of the SARS-CoV E protein play a major role in virulence, its ion channel activity and the PDZ-binding motif (PBM) (15, 16). CoV E protein is also essential for intracellular virus trafficking (17).

CoVs have several proteins that include PBMs that may modify cell behavior to benefit the virus. PBMs are specific sequences usually located at the C terminus of proteins, normally within the last four residues and interact with PDZ domains (an acronym formed by the combination of the first letter of the names of the three first proteins where this domain was identified: PSD-95, Discs large 1, and Zonula occludens 1, respectively), consisting of around 80 to 90 amino acids (aa). There are 266 PDZ domains present within more than 400 protein isoforms in the human genome (18). Three different classes of PBMs are recognized based on the nature of their core sequence motifs: class I PBMs consist of X-S/T-X-Φ_-COOH_, where X can be any amino acid and Φ is a hydrophobic amino acid (normally V, I, or L), class II PBMs are X-Φ-X-Φ_-COOH_, and class III PBMs are X-D/E-X-Φ_-COOH_. Due to the functional versatility of proteins containing PDZ domains, viruses can modify a wide range of cellular functions through their PBMs to enhance viral replication and dissemination, and influence disease pathogenesis (19). Although the E proteins of all hCoVs have a PBM in their C terminus, the PBM of SARS-CoV E protein is the only one that has been studied in detail. The role of E protein PBMs from other hCoVs in virulence remains unknown (15).

SARS-CoV E protein is a small integral membrane protein of 76 aa, including the PBM formed by the last four aa of the carboxy terminus (-DLLV_-COOH_). An identical PBM sequence is present in the SARS-CoV-2 E protein. The SARS-CoV E protein PBM is a known virulence factor (15), inducing an exaggerated proinflammatory response by binding to the host PDZ-containing protein syntenin-1 and possibly to additional host proteins. The interaction of SARS-CoV E protein PBM and syntenin-1 leads to acute respiratory distress syndrome, pulmonary edema, and death at least through the phosphorylation and activation of p38-MAPK, a protein regulating the expression of proinflammatory cytokines (15).

Here, we use reverse genetics to generate variants of SARS-CoV, MERS-CoV, and SARS-CoV-2 that lack the E protein PBM to investigate their pathogenicity in mice. We found that the PBMs of these three hCoVs are virulence factors required for optimal replication and the generation of alveolar edema. Furthermore, we constructed a collection of SARS-CoV mutants in which the E protein PBM was replaced with one derived from virulent or attenuated hCoVs and studied their virulence. A gradient of virulence was observed, depending on whether the E protein was derived from an attenuated or virulent hCoV. Following infection of mice with SARS-CoV, we performed transcript profiling in lung tissues using RNA sequencing (RNA-seq). These studies showed that the E protein PBMs of SARS-CoV or SARS-CoV-2 altered the expression of genes involved in ion transport and cell homeostasis. Of note, we observed decreased expression of cystic fibrosis transmembrane conductance regulator (*CFTR*) mRNA, encoding a protein involved in liquid absorption in the alveolus. The reduction in *CFTR* transcript abundance was associated with increased alveolar edema in the lungs of mice infected with SARS-CoV or SARS-CoV-2. We further investigated the effects of small molecule CFTR modulators on the replication of SARS-CoV-2 in cultured cells and observed that these compounds significantly reduced SARS-CoV-2 replication and protected against severe disease in a mouse model. These results demonstrate the importance of the E protein PBMs in CoV replication and virulence and identify novel cellular targets for the selection of antivirals.

## RESULTS

### Relevance of E protein PBM from virulent hCoVs on viral replication *in vitro* and *in vivo*.

**FIG 1:**
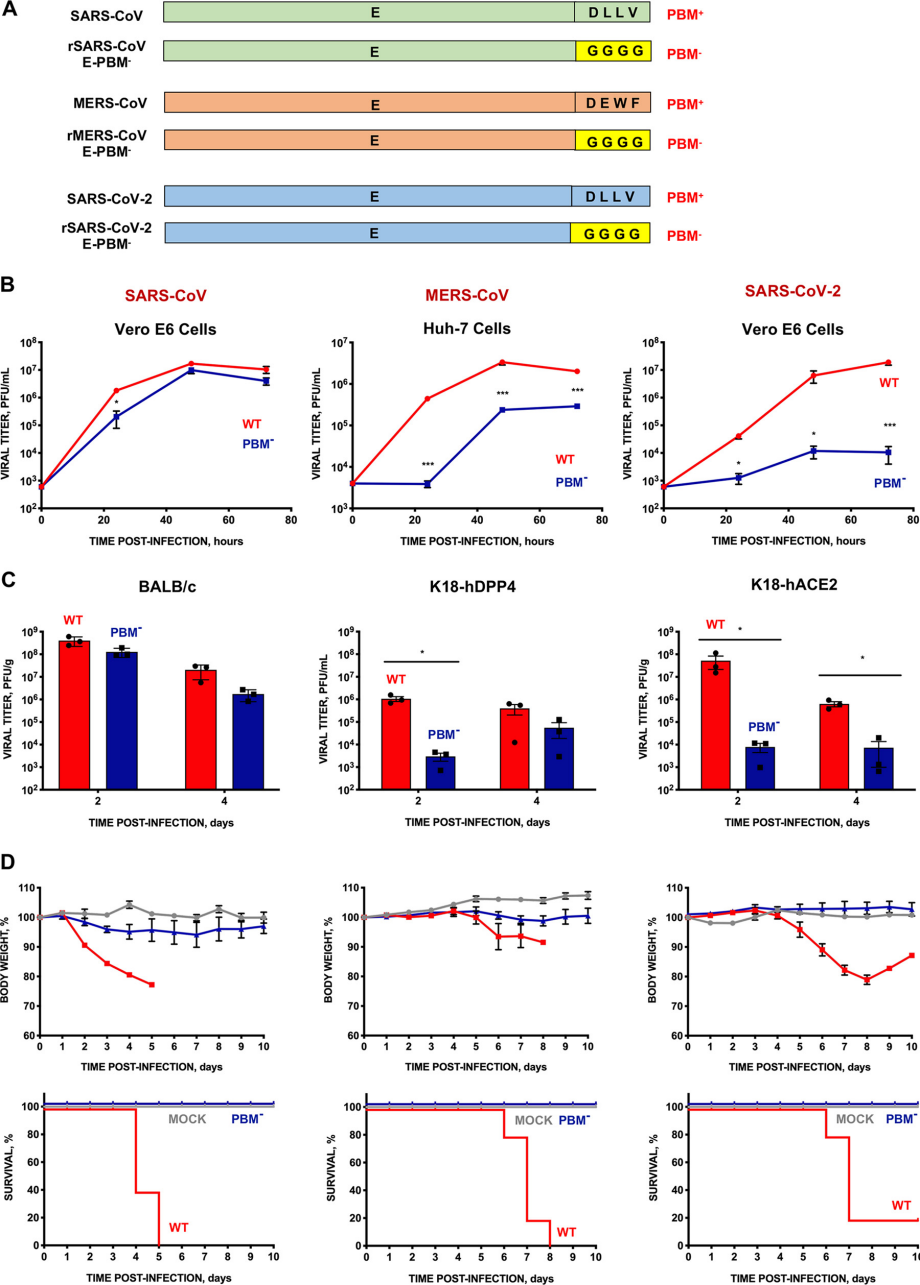
Growth and virulence of SARS-CoV, MERS-CoV, and SARS-CoV-2 mutants lacking E protein PBM. (A) Diagram mutant pairs, with (PBM^+^) and without the PBM (PBM^−^), were generated for each of the three viruses. In the rSARS-CoV-E-PBM^−^, rMERS-CoV-E-PBM^−^, and rSARS-CoV-2-E-PBM^−^ mutants, the PBM of E protein was replaced by four glycines. (B) Subconfluent Vero E6 or Huh-7 cells were infected with an MOI of 0.001 with each of the three viruses. Wild- type (WT) viruses are shown with red lines and symbols. Mutants without E protein PBM (PBM^−^), with blue lines and symbols. Supernatants were collected at 24, 48, and 72 hpi and titrated by the lysis plaque formation method. (C) Groups of six 16-week-old BALB/c, K18-hDPP4, or K18-hACE2 mice were intranasally inoculated with 10,000 PFU of the WT viruses (red columns) or each of the mutants lacking E protein PBM (blue columns). Three mice from each group were euthanized at 2 and 4 dpi to analyze virus production in lung. Vertical bars represent the standard error of the mean. Statistically significant data are represented according to the *P* value obtained in Student’s *t* test analysis: *, *P < *0.05; ***, *P* < 0.001. (D) Groups of five 16-week-old BALB/c, K18-hDPP4, or K18-hACE2 mice were mock-infected (gray lines) or intranasally inoculated with 10,000 PFU with each of the parental (WT) virus (red lines), or with their corresponding mutants without PBM of E protein (PBM^−^) (blue lines). Weight loss (top) and survival (bottom) of mice were monitored for 10 days postinfection. Vertical bars represent the standard error of the mouse weight mean.

Using an infectious cDNA clone engineered in our laboratory, we constructed a collection of mutant viruses by replacing the 4 residues of the PBM core with glycines (Fig. 1A). The growth kinetics of mouse-adapted SARS-CoV and SARS-CoV-2 wild-type and deletion mutants were assessed in Vero E6 cells. Similar studies of modified MERS-CoV were performed in Huh-7 cells. While the growth of rSARS-CoV with or without E PBM was similar, the E PBM deletion mutants of SARS-CoV-2 and MERS-CoV exhibited between 10 and 1,000-fold lower viral titers, respectively, than the parental (wt) viruses (Fig. 1B). This suggests that SARS-CoV-2 and MERS-CoV required an E PBM for optimal virus replication in cultured cells.

To investigate the role of E protein PBM in virus replication *in vivo*, BALB/c, K18-hDPP4, or K18-hACE2 mice were infected with either wild-type (wt) SARS-CoV, MERS-CoV, or SARS-CoV-2, respectively, or their corresponding mutants lacking E PBMs. Lung tissue titers were determined at days 2 and 4 postinfection (dpi) (Fig. 1C). The MERS-CoV and SARS-CoV-2 E protein deletion mutants grew to 1,000- to 10,000-fold lower titers that the corresponding parental viruses, indicating that E protein PBMs contributed to efficient virus replication *in vitro* and *in vivo*. We then monitored weight loss and survival through 10 dpi. All mice infected with parental viruses lost weight and exhibited mortality ranging from 80% to 100%, as expected (Fig. 1D). In contrast, all mice infected with virus lacking E protein PBM expression survived, further supporting the conclusion that the E protein PBM is a CoV virulence factor.

**FIG 2:**
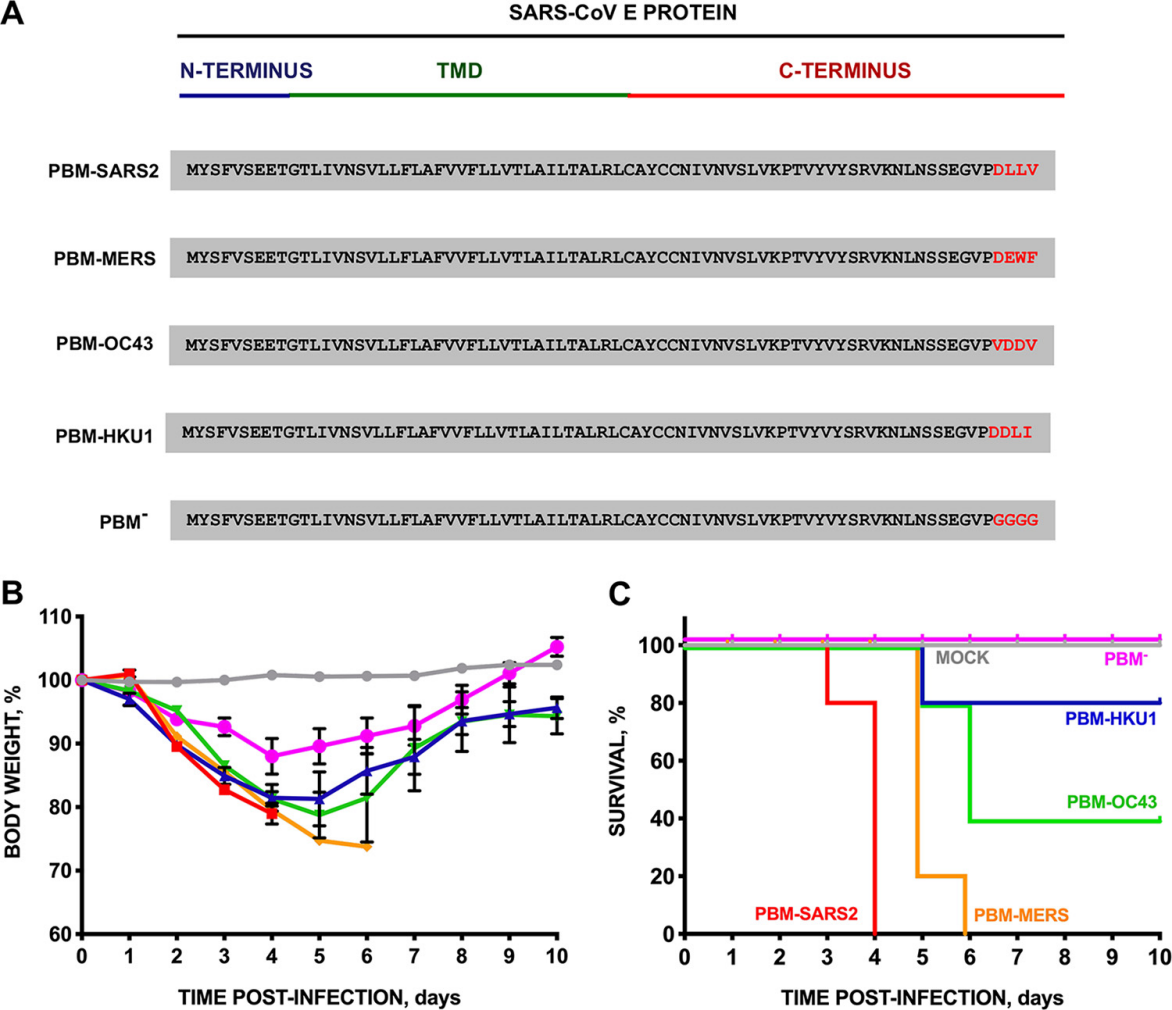
Virulence of SARS-CoV mutants with the PBM of E protein from attenuated or virulent β-hCoVs. (A) E protein sequence of parental and the corresponding mutants in which the PBM of E protein was replaced by that of an alternative virulent or attenuated β-hCoV (in red). Groups of five 16-week-old BALB/c mice were inoculated intranasally with DMEM (uninfected, gray), or with 100,000 PFU of parental SARS-CoV (PBM-SARS2), or with the generated mutants: PBM-MERS (orange line), PBM-OC43 (green line), and PBM-HKU1 (dark blue line), and PBM^−^ (pink line). (B and C) Weight loss (B) and survival (C) were monitored for 10 dpi. Vertical bars represent the standard error of the mean weight of the mice. TMD, transmembrane domain.

Human CoVs cause mild to severe respiratory disease. To assess whether disease severity is influenced by the source of the PBM, we engineered a set of recombinant SARS-CoVs expressing the E protein PBM sequences of virulent or attenuated β-hCoV, including SARS-CoV-2, MERS-CoV, HCoV-OC43, and HCoV-HKU1 (Fig. 2A). As a control, a rSARS-CoV without a PBM core sequence was generated (rSARS-PBM^−^). We inoculated BALB/c mice intranasally with the recombinant viruses and monitored weight loss (Fig. 2B) and survival (Fig. 2C) for 10 dpi. While all mock-infected mice and mice infected with SARS-CoV lacking E protein PBM survived, mice infected with recombinant viruses containing the parental virus PBM from SARS-CoV-2 (PBM-SARS2) or MERS-CoV (PBM-MERS) lost weight and died between 4 and 6 dpi, respectively. Interestingly, we observed a gradient of lethality among these highly virulent viruses. All mice infected with PBM-SARS2 died with less than 20% weight loss. In contrast, all mice infected with PBM-MERS exhibited more than 25% weight loss and required euthanasia. Mice infected with viruses containing the E protein PBM core sequences from the attenuated hCoVs HKU1 and OC43 demonstrated intermediate survival phenotypes with 40% and 80% survival, respectively. Thus, substituting the SARS-CoV E protein PBM core sequence with those from other hCoVs altered virulence. Lung tissue virus titers from SARS-CoV with native or modified PBMs were similar, indicating that the observed differences in virulence were not due to changes in virus titers (Fig. S1 in the supplemental material).

10.1128/mbio.03136-22.1 FIG S1 Growth kinetics of SARS-CoV E protein PBM mutants. (A) Subconfluent Vero E6 were infected with SARS-CoV E protein PBM mutants at a MOI of 0.001. The cells infected with different virus mutants are indicated with lines of different color: native viruses (wt) are shown with red lines and the mutants without E protein PBM (PBM^−^) with pink lines and symbols. PBM-MERS-CoV (orange lines), PBM-hCoV-OC43 (green lines), and PBM-hCoV-HKU1 (blue lines). Supernatants were collected at 24, 48, and 72 hpi and titrated by the lysis plate formation method. (B) Groups of six 16-week-old BALB/c mice were intranasally inoculated with 100,000 pfu of the native viruses (red columns), or PBM^−^ (pink column), PBM-MERS-CoV (orange column), PBM-OC43 (green column), and PBM-HKU1 (blue column). Three mice from each group were euthanized at 2 and 4 dpi to analyze virus production in lungs. Vertical bars represent the standard error of the mean titers obtained by repeating the experiment three times. Statistically significant data are represented according to the *P* value obtained in Student’s *t* analysis: **, *P < *0.01. Download FIG S1, TIF file, 0.3 MB. Copyright © 2023 Honrubia et al. 2023 Honrubia et al. https://creativecommons.org/licenses/by/4.0/ This content is distributed under the terms of the Creative Commons Attribution 4.0 International license.

### E protein PBM conditions β-hCoV pathogenesis.

**FIG 3:**
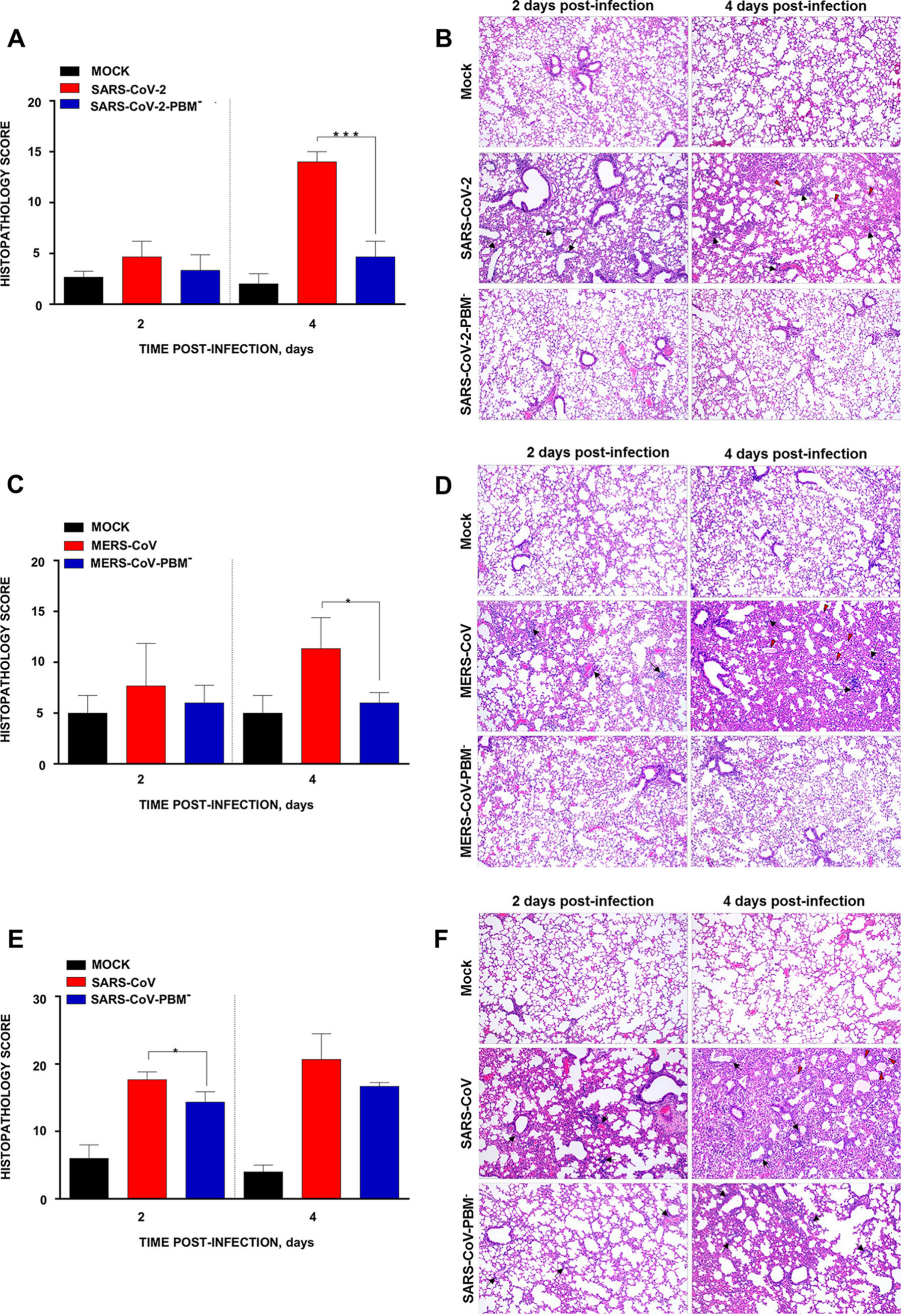
Lung pathology associated with E protein PBM of SARS-CoV, MERS-CoV, and SARS-CoV-2. Lung histopathology scores were examined in lung samples taken from K18-hACE2 (A), K18-hDPP2 (C), and BALB/c mice (E) infected with SARS-CoV-2, MERS-CoV, and SARS-CoV parental and mutant viruses, respectively, as indicated in Materials and Methods (*n* = 3/group), and euthanized at day 2 and 4 postinfection (dpi). Representative lung histopathological sections (H&E staining) from infected K18-hACE2 (B), K18-hDPP4 (D), and BALB/c (F) mice are shown (magnification: 10×). Mean and SD of cumulative histopathological lesion scores are represented. Unpaired *t* test: *, *P < *0.05; ***, *P < *0.001.

At 2 dpi, SARS-CoV-2-infected K18-hACE2 mice displayed mild alveolar septal thickening and mild perivascular and peribronchiolar mononuclear infiltrates. These lesions increased by 4 dpi, and mice also developed alveolar edema (Fig. 3A and B) with a histopathology score of 14.0. However, mice infected with the mutant SARS-CoV-2-PBM^−^ exhibited no significant inflammatory or vascular changes having a score of 4.7. K18-hDPP4 mice infected with MERS-CoV showed moderate lung inflammatory lesions at 2 dpi that increased in severity by day 4 pi with a score of 11.4, including the presence of diffuse alveolar septal thickening, and moderate perivascular and peribronchiolar mononuclear infiltrates. In contrast, mice infected with the mutant MERS-CoV-PBM^−^ showed a score of around 6.0 with no significant inflammatory lesions at either time point (Fig. 3C and D). In BALB/c mice infected with SARS-CoV, lung tissue pathology was more severe than in BALB/c mice infected with the SARS-CoV-PBM^−^ mutant with histopathology scores of 17.7 and 13.5 at 2 dpi, respectively. These differences included severe interstitial pneumonia, perivascular and peribronchiolar mononuclear infiltrates, and alveolar edema (Fig. 3E and F).

**FIG 4:**
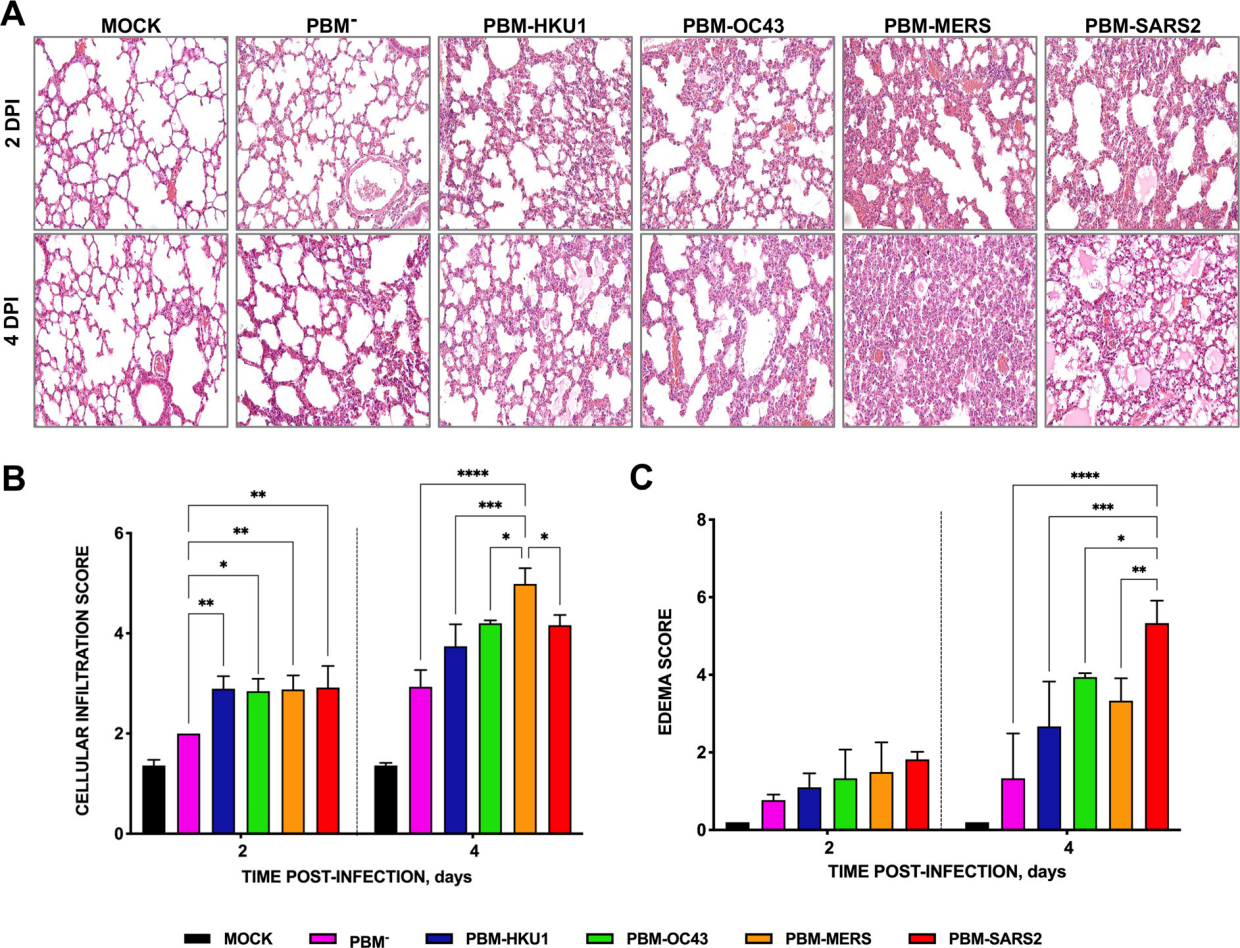
Lung pathology in SARS-CoV-infected mice due to the presence of E protein PBM from attenuated or virulent β-hCoVs. Groups of six 16-week-old mice were mock infected or inoculated with 100,000 PFU of a SARS-CoV including E proteins with a PBM derived either from the SARS/SARS-2-CoV, or from MERS-CoV, hCoV-OC43, hCoV-HKU1, or with the PBM replaced by four glycines (PBM^−^). Three mice from each group were euthanized, and lungs were collected at 2 and 4 dpi. Lungs were fixed in formalin with zinc, embedded in kerosene, and cut and stained with hematoxylin and eosin. Cellular infiltration (A) and edema (B) scores were determined in lung samples as indicated in Materials and Methods (*n* = 3/group). The vertical bars represent the mean and the standard deviation of the data obtained in the lung of each of the three mice used for each experimental setting. Unpaired *t* test: *, *P < *0.05; **, *P < *0.01; ***, *P < *0.001; ****, *P* < 0.0001.

We performed additional histologic analysis of lung tissue sections from mice infected with rSARS-CoVs with PBMs from attenuated or virulent CoVs at 2 or 4 dpi to assess immune cell infiltration and alveolar edema. Mice infected with virus lacking the E protein PBM core sequence (PBM^−^) showed minimal inflammatory cell infiltrates and edema only at 4 dpi (Fig. 4). Mice infected with rSARS-CoV with an E protein PBM from HCoV-HKU1 (PBM-HKU1) or HCoV-OC43 (PBM-OC43) developed significant cellular infiltrates at 2 dpi that persisted at 4 dpi, while mice infected with virulent SARS-CoVs showed progressive infiltrates at the same time points (Fig. 4). Mice infected with viruses encoding the E protein PBM core from SARS-CoV-2 showed moderate interstitial and peribronchiolar cell infiltration at 2 dpi and more alveolar edema at 4 dpi coincident with the greater lethality of this virus. Interestingly, mice infected with recombinant viruses encoding an E protein PBM core from MERS-CoV showed a phenotype similar to that observed for PBM-SARS2 at 2 dpi but with a significant increase in cell infiltration and alveolar and bronchiolar wall thickening at 4 dpi, compared to mice infected with viruses encoding E PBMs from attenuated CoVs (Fig. 4). These data indicated that virus virulence correlated with lung tissue pathology and the E protein PBM core sequence contributed to these findings.

### Effect of E protein PBM virus origin on host gene expression.

**FIG 5:**
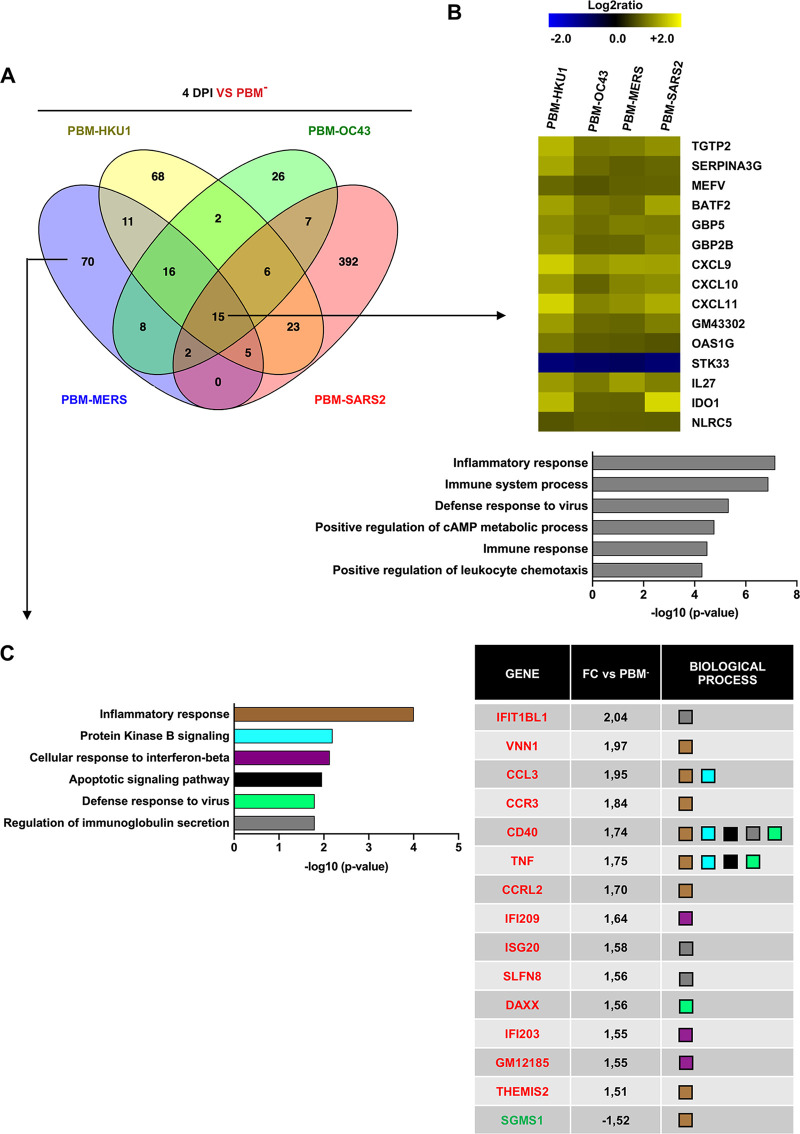
Differential and common gene expression patterns among the different rSARS-CoV with E protein PBM from virulent or attenuated β-hCoVs. (A) Venn diagram of genes differentially expressed at least 1.5-fold with an FDR <0.05 in the lungs of mice infected with the different rSARS-CoVs with the PBMs from the indicated hCoVs, compared to rSARS-CoV without the E protein PBM (PBM^−^), at 4 dpi. (B) Expression profile of the most frequently expressed genes due to the presence of a PBM from an attenuated or virulent hCoV. Analysis of the 15 genes with highest variation, due to the presence of E protein specific PBMs. The clustering of genes based on the biological process terms defined in Gene Ontology are shown. The six most relevant biological processes at 4 dpi into which the 15 commonly expressed genes are classified, have been listed. The values on the *x* axis indicate the −log_10_ (*P* value) values obtained with the DAVID software. (C) Biological activity types into which the 70 genes that are differentially expressed in the lungs of rSARS-CoV-PBM-MERS-infected mice are grouped, compared to rSARS-CoVs without PBM or with PBMs from other hCoVs. The grouping of these genes into distinct sets is based on the biological processes defined in Gene Ontology. The six most relevant biological processes are described. A table with the fold change (FC) and the biological processes in which genes of the six most relevant biological processes are involved is provided. The names of the genes in red and green correspond to those that increase or decrease their expression, respectively.

To investigate the virulence mechanisms of rSARS-CoV with PBMs from the different hCoVs, we performed RNA-seq analysis on lung tissue from infected mice. Because the observed association of virulence and lung histopathology scores was greater at 4 dpi, we focused on this time point. A Venn diagram of differentially expressed genes (DEGs) with a fold change (FC) higher than 1.5 and a false discovery rate (FDR) lower than 0.05 (|FC|>1.5; FDR <0.05, respectively) of mice infected with rSARS-CoVs with PBMs from hCoVs causing a range of pathology compared to the PBM^−^ mutant was determined (Fig. 5A). We sought to identify transcripts with expression driven by the presence of an E protein PBM from attenuated or virulent β-hCoVs. The presence of an E protein PBM from attenuated β-hCoVs reduced SARS-CoV mortality (Fig. 2B and C). A set of 14 transcripts that increased their abundance after infection with all four viruses, including PBM-HKU1, PBM-OC43, PBM-MERS, or PBM-SARS2, was upregulated in all cases, whereas 1 (STK33) was downregulated in comparison with the infection by a SARS-CoV including a nonfunctional PBM that was replaced by four glycines (Fig. 5B). These transcripts were classified using the Gene Ontology analysis (DAVID software) as mainly related to immune and inflammatory responses (Fig. 5B), suggesting that the presence of a functional E protein PBM from either an attenuated or virulent β-hCoV triggered a host innate immune response.

Replacing the SARS-CoV E protein PBM core with the homologous sequence of a virulent hCoV (PBM-MERS or PBM-SARS2) increased lung injury and virus pathogenicity compared to replacement by the PBM core from an attenuated hCoV (PBM-HKU1 and PBM-OC43). We next focused on the 70 and 392 DEGs that were solely changed in expression by viruses encoding the E protein PBM from MERS-CoV or SARS-CoV-2 at 4 dpi, respectively (Fig. 5A). The 70 DEGs enriched in tissues from mice infected with CoVs encoding a PBM-MERS were associated with host immune and inflammatory responses (Fig. 5C).

**FIG 6:**
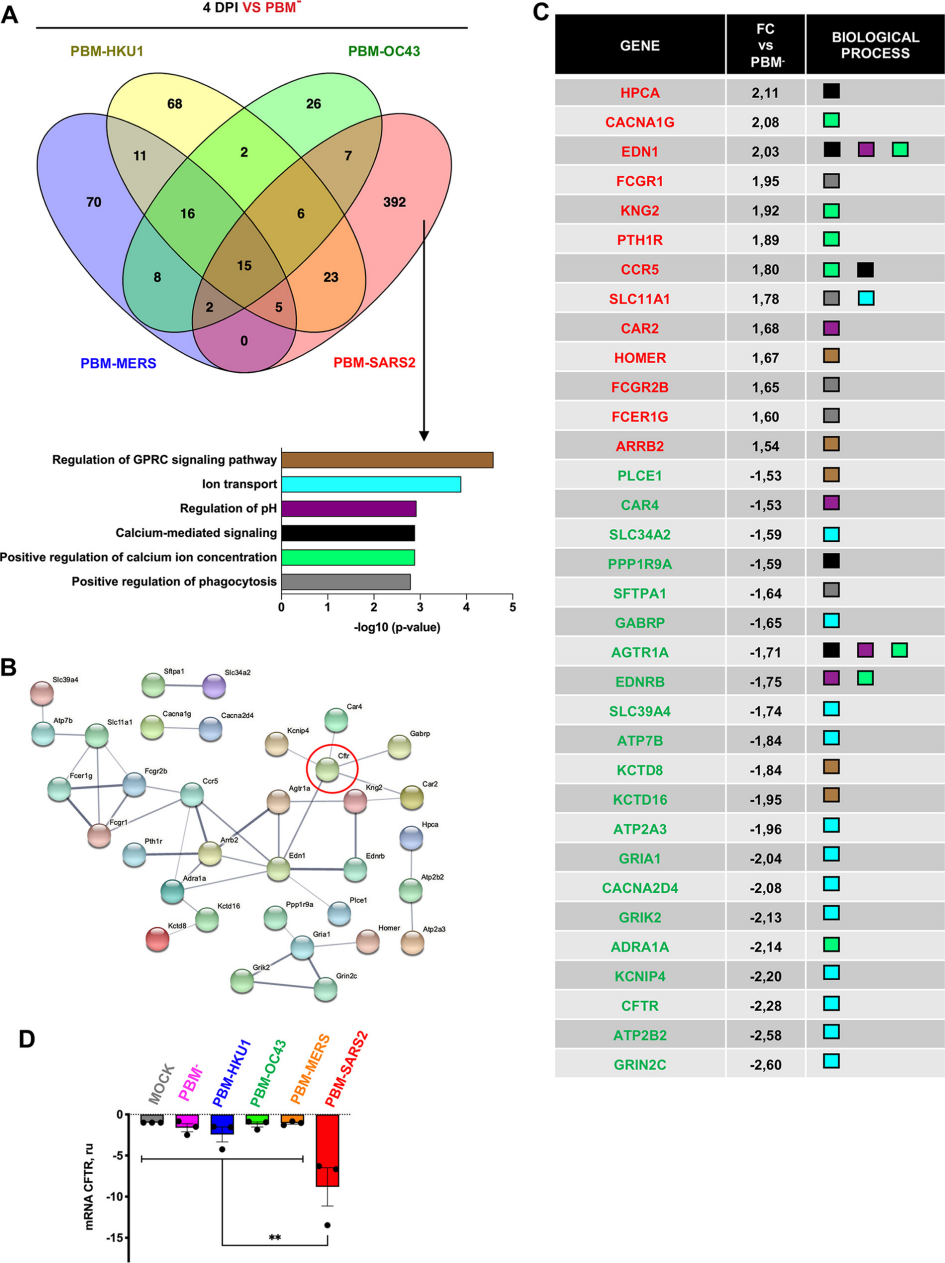
Biological activity groups of genes differentially expressed exclusively due to the presence of E protein PBM from either SARS-CoV and SARS-CoV-2. (A) Clustering of 392 genes that are differentially expressed in the lungs of mice infected with the native virus compared to rSARS-CoVs without a PBM or with a PBM from another hCoV. The clustering of these genes is based on the biological process terms defined in Gene Ontology. The six more relevant biological processes are represented. (B) Network of interactions between genes that belong to the most relevant biological processes according to the STRING database. The thickness of the lines indicates the robustness of the data supporting each interaction and all those interactions with a score >0.4 were considered statistically significant. (C) Table with information on the rate of change (FC) and the biological processes to which the genes of the most relevant interaction networks belong. The names of the genes are shown in red or in green depending on whether correspond to those that increased or decreased their expression. (D) RT-qPCR of CFTR mRNA expression in lungs from mice infected with SARS-CoV E protein PBM mutants. RNA from lungs of mock-infected mice (gray column)or infected with PBM-SARS2 (red column) or with the E protein PBM mutants PBM-MERS (orange column), PBM-HKU1 (blue column), PBM-OC43 (green column), and PBM^−^ (pink column) were quantified by RT-qPCR at 4 dpi. Mean values and their standard deviations are presented. Statistically significant data are indicated according to the *P* value obtained in Student’s *t* analysis: **, *P* < 0.01.

**FIG 7:**
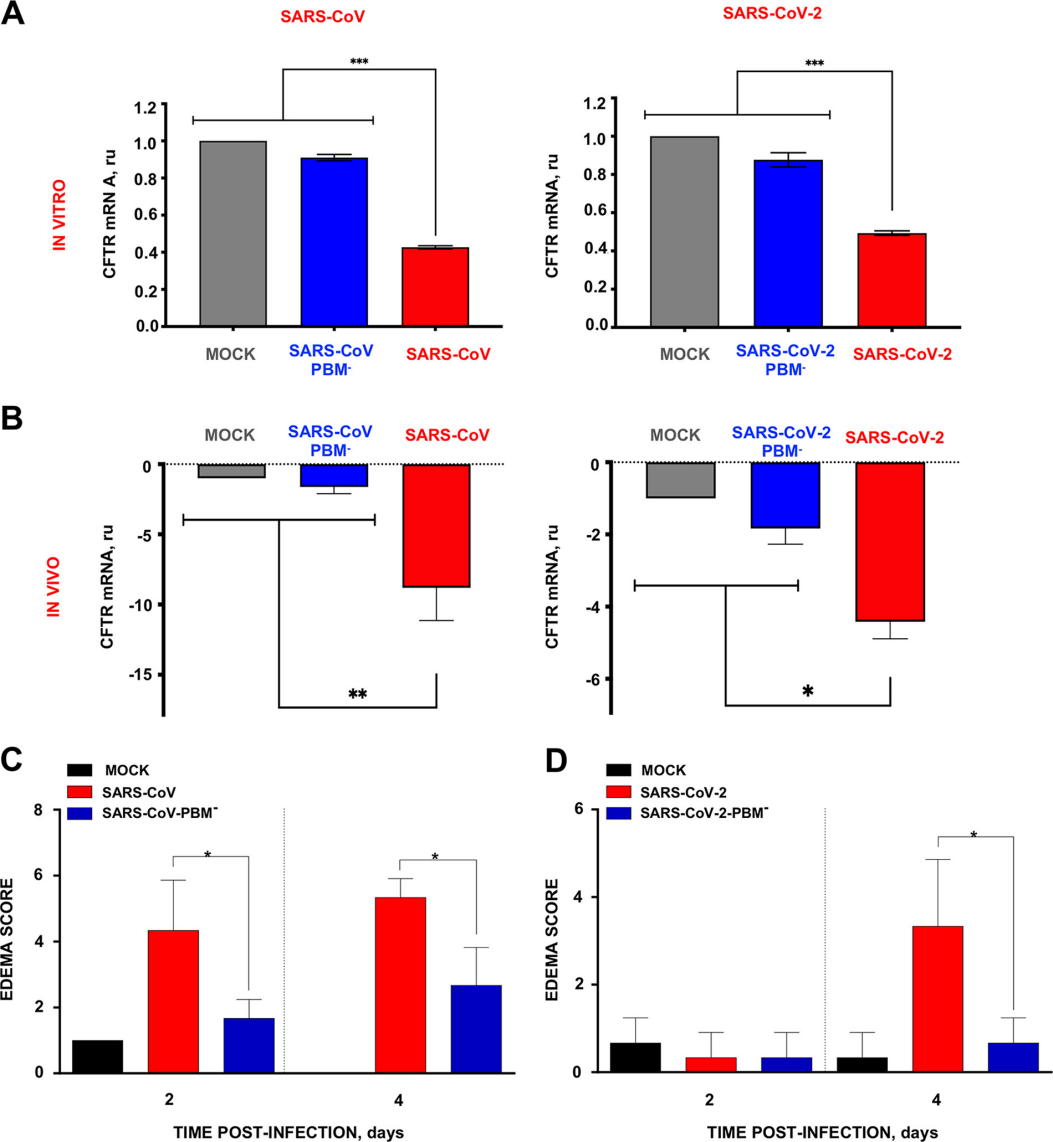
CFTR mRNA expression decreased during SARS-CoV and SARS-CoV-2 infection in the presence of E protein PBM. (A and B) CFTR mRNA expression in Calu-3 cells (A) and lungs (B) from mice infected with SARS-CoV or SARS-CoV-2 with and without E protein PBM was analyzed by qPCR at 48 hpi and 4 dpi, respectively. (C and D) Edema scores examined in lung samples taken from K18-hACE2 or BALB/c mice infected with SARS-CoV-2 or SARS-CoV parental or mutant viruses, respectively, as indicated in Materials and Methods (*n* = 3/group), and euthanized at 2 and 4 days postinfection (dpi). Mean and SD of cumulative histopathological lesion scores. Unpaired *t* test: *, *P < *0.05; **, *P < *0.01; ***, *P < *0.001.

Among the 392 DEGs enriched during infection with viruses containing the E PBM from SARS-CoV or SARS-CoV-2 (Fig. 6A), 52 were present within biological activity processes related to ion transport and homeostasis (Fig. 6A). At least 34 of these 52 transcripts were identified in a highly interactive network (Fig. 6B). The genes with increased expression were mainly associated with the regulation of cytosolic Ca^2+^ concentration while those with decreased expression were associated with ion transport and cell homeostasis (Fig. 6C). These transcriptional changes were associated with greater pulmonary edema scores and mortality. Among these genes we noted that *CFTR* transcript abundance was reduced only in the presence of E protein PBM from SARS-CoV and SARS-CoV-2. The reduction in *CFTR* mRNA expression in the lungs of mice infected with SARS-CoV, containing a PBM identical to that of SARS-CoV-2, was confirmed by quantitative PCR at 4 dpi (Fig. 6D). SARS-CoV and SARS-CoV-2 infection reduced *CFTR* mRNA transcript abundance in Calu-3 2b4 cells and lung tissue of infected mice (Fig. 7A and B).

### CFTR modulators modify virus replication in infected cells.

In the alveolus, CFTR plays an important role in fluid absorption and thus loss of CFTR function could contribute to alveolar edema (20–23). Consistent with this, mice infected with SARS-CoV-2 or SARS-CoV had more alveolar edema compared to the PBM-variants (Fig. 7C and D). Recently, small molecule CFTR modulators were discovered that improve the function of some mutant forms of CFTR protein associated with cystic fibrosis. The corrector compound VX809 (lumacaftor) acts as a chemical chaperone and partially rescues CFTR function in cells expressing the misfolded F508del CFTR protein (24, 25). The potentiator VX770 (ivacaftor) increases the probability of the CFTR anion channel associated with G551D and related conductance mutations being in an open conformation (26–30). Of note, these CFTR modulators also enhance the function of wt CFTR (31–33).

**FIG 8:**
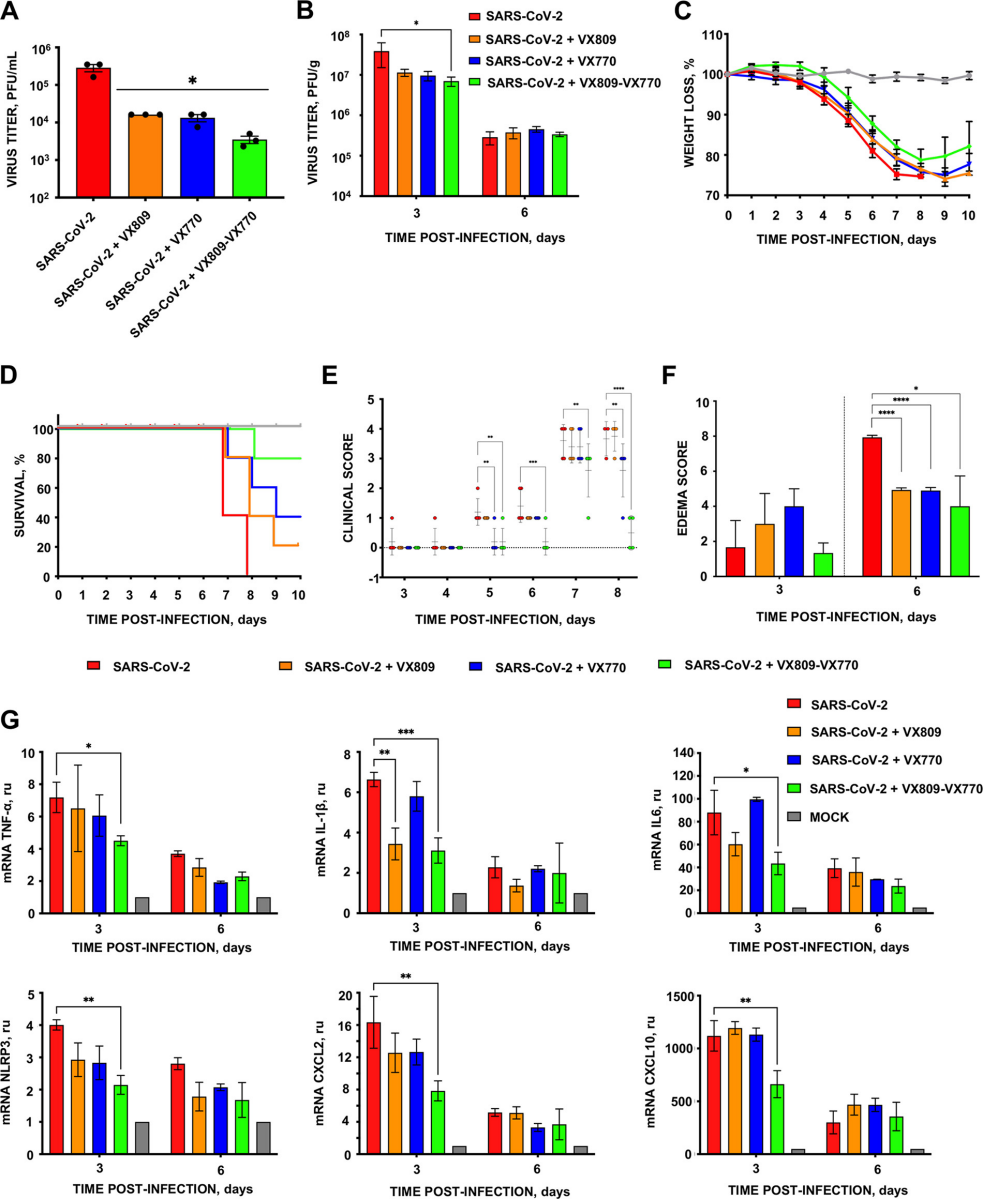
Protection of mice infected with SARS-CoV-2 by the modulation of CFTR expression. (A) Subconfluent Calu-3 2B4 cells were infected (MOI of 0.1) with SARS-CoV-2 wt virus. Infected cells were nontreated (red lines) or incubated with lumacaftor (VX809; 50 μM) (orange column), ivacaftor (VX770; 5 μM) (blue column) or both (green column). Culture supernatants were collected at 48 hpi and extracellular virus was titrated by the lysis plaque formation method. (B) Groups of five 20-week-old K18-hACE2 mice were intranasally inoculated with 10,000 PFU and daily intraperitoneally inoculated until 8 dpi with lumacaftor (12 mg/kg; orange column), ivacaftor (6 mg/kg; blue column), or both (green column). Three mice from each group were euthanized at 3 and 6 dpi to analyze virus production in lung. Vertical bars represent the standard error of the mean titers obtained by repeating the experiment three times. Statistically significant data are represented according to the *P* value obtained in Student’s *t* test: *, *P < *0.05. Weight loss (C) and survival (D) of mice were monitored for 10 days postinfection. Vertical bars represent the standard error of the mouse mean weight. (E) Clinical score of mice infected with SARS-CoV-2 in the absence (red circle) or presence of VX809 (orange circle), VX770 (blue circle), or both CFTR modulators (green circle). Five mice per group were monitored daily for 10 dpi and their clinical signs of disease were classified in five grades of severity. Vertical bars represent the standard deviation of the mean derived from clinical scores obtained from five mice. Statistically significant data are represented according to the *P* value obtained in Student’s *t* analysis: **, *P < *0.01; ***, *P* < 0.001; ****, *P* < 0.0001. (F) Edema scores were determined in lung samples as indicated in Materials and Methods (*n* = 3/group). The vertical bars represent the mean and the standard deviation of the data obtained in the lung of each of the three mice used for each experimental setting. Unpaired *t* test: *, *P < *0.05; ****, *P* < 0.0001. (G) mRNA quantification by RT-qPCR of inflammation mediators at 3 and 6 dpi in the lungs of infected mice treated with lumacaftor and ivacaftor. mRNA levels were compared to those in mock-infected mice, using the 2^−ΔΔ^*^CT^* method and the 18S rRNA as the normalization endogenous control. Error bars indicate the standard error of the mean. Statistical significance was calculated by two-tailed Student’s *t* test. *, *P* < 0.05; **, *P* < 0.01; ***,*P* < 0.001.

We hypothesized that CFTR modulator treatment would partially rescue CFTR function and modify the disease phenotype. To test this hypothesis, CFTR modulators VX809 and VX770 were administered to Calu-3-2B4 cells and to K18-hACE2 mice infected with SARS-CoV-2. SARS-CoV-2 titers were measured at 48 h postinfection in cell culture, the time point at which the highest titer is reached (Fig. S2). CFTR modulator treatment resulted in a 10- and 7-fold reduction in virus titers *in vitro* and *in vivo*, respectively, in relation to nontreated cells or mice (Fig. 8A and B). At these concentrations, the modulators were nontoxic to Calu-3-2B4 cells (Fig. S3). Remarkably, the combined treatment of mice infected with SARS-CoV-2 with the VX809 and VX770 was associated with 80% survival, while mock-treated animals had 100% mortality. Still, a minimal effect on weight loss was observed, which is in line with previous observations that weight loss is a more sensitive measure of virus pathogenesis than survival (reference 15). Mice receiving VX770 or VX809 alone exhibited 40% and 20% survival, respectively (Fig. 8C and D). Treatment with the combination of both CFTR modulators delayed the appearance of clinical signs due to SARS-CoV-2 infection. SARS-CoV-2-infected and nontreated mice showed the highest clinical score (3.6) at both 7 and 8 dpi. In contrast, infected mice treated with both CFTR modulators showed a clinical score of 2.6 and 0.5 at the same times, respectively (Fig. 8E). Moreover, infected mice treated with CFTR modulators individually showed less edema score (4.9 for both cases) at 6 dpi in comparison with untreated mice (7.9). The combination of both CFTR modulators also reduced the edema score (4.0) (Fig. 8F).

10.1128/mbio.03136-22.2 FIG S2 Growth kinetics of SARS-CoV-2 in Calu-3 2B4. Subconfluent Calu-3 2B4 cells were infected with SARS-CoV-2 parental virus (red line) at a MOI of 0.1. Supernatants were collected at 24, 48 and 72 hpi and titrated. Vertical bars represent the standard error of the mean. Download FIG S2, TIF file, 0.1 MB. Copyright © 2023 Honrubia et al. 2023 Honrubia et al. https://creativecommons.org/licenses/by/4.0/ This content is distributed under the terms of the Creative Commons Attribution 4.0 International license.

10.1128/mbio.03136-22.3 FIG S3 Viability of Calu-3 2B4 cells in the presence of different CFTR protein modulators. Subconfluent and uninfected Calu-3 2B4 cells were incubated with different concentrations (1 to 50 μM) of ivacaftor (A) and lumacaftor (B) and with the combination of ivacaftor (5 μM) and lumacaftor (50 μM) (green column) (C). Supernatants were collected at 48 hpi and cell viability was determined by the MTT [3-(4,5-dimethylthiazol-2-yl)-2,5-diphenyltetrazole bromide] reduction assay (88). Mean values of cell viability and their standard deviations are shown. Download FIG S3, TIF file, 0.2 MB. Copyright © 2023 Honrubia et al. 2023 Honrubia et al. https://creativecommons.org/licenses/by/4.0/ This content is distributed under the terms of the Creative Commons Attribution 4.0 International license.

### Effect of CFTR modulators on inflammatory cytokines induced by SARS-CoV-2 in the lungs of infected mice.

SARS-CoV-2 E protein modulates the NLRP3 inflammasome (34). CFTR modulators reduced NLRP3 inflammasome-mediated activation in monocytes derived from CF patients (35). We investigated the effects of CFTR modulators on NLRP3 mRNA transcript abundance and NLRP3-dependent cytokines (TNF-α, IL-6, and IL-1β) and chemokines (CXCL10 and CXCL2) in the lung tissues of SARS-CoV-2-infected K18-hACE2 mice by qRT-PCR (Fig. 8G) (36–38). SARS-CoV-2 infection increased the expression of NLRP3 cytokines and chemokines at 3 dpi. In contrast, the lungs of CFTR modulator-treated mice infected with SARS-CoV-2 showed lower levels of NLRP3 and NLRP3-dependent cytokines and chemokines (Fig. 8G) with more obvious effects when both modulators were used. These results suggest that CFTR modulators might also have an anti-inflammatory effect during SARS-CoV-2 infection (39–41).

## DISCUSSION

### Relevance of E protein PBM core motifs as virulence determinants.

In this article, the role of the E protein PBMs in human CoV replication and virulence is investigated, leading to the discovery that E protein PBM from SARS-CoV and SARS-CoV-2 reduces the transcript abundance for several genes involved in ion transport and cellular homeostasis (Fig. 6A), including CFTR (Fig. 6B and C). It is also shown that the combination of two drugs previously used to treat cystic fibrosis increased by 80% the survival of humanized mice after their infection by SARS-CoV-2.

Using reverse genetics, we generated a collection of SARS-CoV mutants by replacing its E protein PBM with homologous PBMs from attenuated or virulent β-hCoVs (SARS-CoV-2, MERS-CoV, hCoV-OC43, and hCoV-HKU1). The engineered mutants showed significant differences in virulence in mice. The PBMs from common cold hCoVs partially attenuated SARS-CoV and showed less cellular infiltrates and alveolar edema, compared to mice infected with the parental virus (Fig. 4). In contrast, rSARS-CoVs with PBM cores from virulent hCoVs caused the death of all infected mice and were associated with diffuse cellular infiltrates and alveolar edema. While only a small number of viruses were analyzed, these results support the notion that the PBMs play a critical role in virus virulence. Analysis of additional CoVs should be performed to confirm (or not) this interesting association.

SARS-CoV and SARS-CoV-2 share identical E protein PBMs. Infection of mice with these two viruses caused the greatest alveolar edema and mortality. In contrast, the introduction of the MERS-CoV PBM core was associated with increased cellular infiltrates and alveolar wall thickening. These results suggest that the E protein PBMs from all three virulent human CoVs are drivers of virus pathogenicity. PBMs encoded by viral proteins have identified roles in the virulence of other viruses. For example, The NS1 protein of the influenza virus also contains a PBM in its terminal carboxyl that is involved in virulence (42, 43). Rabies virus (RABV) strains with different G protein PBMs showed a spectrum of virulence regulated by the activation of different signaling pathways (44, 45).

### Effects of E protein PBMs on host gene expression.

We identified host mRNA transcripts with expression significantly altered by infection with recombinant SARS-CoVs in which only the E protein PBM was modified. The introduction of the MERS-CoV PBM into SARS-CoV maintained expression of inflammatory response-related genes, particularly late in the infection, unlike the PBMs from other virulent viruses (Fig. 5C). For example, high levels of SLFN8, CCL3, CCR3, and CCRL2 were observed in addition to factors related to T-cell proliferation, leukocyte recruitment, eosinophil accumulation, and recruitment of effector immune cells (46–49). Increases in these factors could contribute to the cellular infiltrates observed in the lungs of mice infected with SARS-CoV mutants encoding the MERS-CoV PBM core domain.

We have shown that E protein PBM from SARS-CoV and SARS-CoV-2 reduces the transcript abundance for several genes involved in ion transport and cellular homeostasis (Fig. 6A), including CFTR (Fig. 6B and C).

### Influence of CFTR on the lung pathology produced by SARS-CoV and SARS-CoV-2.

CFTR dysfunction in alveolar epithelial cells impairs alveolar fluid absorption by reducing Cl- permeability across the alveolar epithelium (23). CFTR is required to establish the osmotic gradient for fluid absorption from the alveolar space via aquaporin channels and paracellular pathways (20). Interactions between the SARS-CoV and SARS-CoV-2 E protein PBMs and CFTR may increase alveolar capillary and endothelium permeability, favoring edema formation. CFTR may function in an anti-inflammatory capacity (50–55). The membrane protein (M2) of influenza A has intrinsic ion channel activity and also inhibits CFTR during infection (56). Therefore, it is possible that CFTR inhibition could be a mechanism to induce respiratory virus pathogenesis. In fact, previous studies identified an important role for CFTR in ion and fluid transport across the alveolus. Adenovirus-mediated CFTR expression in rat lungs increased fluid absorption (57), whereas CFTR-deficient animals exhibit impaired alveolar fluid absorption in epithelial cells (58). We found that the CFTR modulators ivacaftor and lumacaftor reduced SARS-CoV-2 replication. Furthermore, infection with native SARS-CoV-2 virus reduced *CFTR* mRNA expression, while a deletion mutant without an E protein PBM did not (Fig. 7A and B) (59). Similar observations were reported using *in vitro* and *in vivo* models of influenza A virus infection (60). Influenza A virus infection reduced CFTR protein abundance and ion channel activity, and these changes were restored by CFTR modulators (60). Ivacaftor also improved alveolar liquid clearance in isolated pig lung lobes *ex vivo* and reduced edema in a volume overload *in vivo* pig model of hydrostatic pulmonary edema (61).

Loss of CFTR function also alters Ca^2+^ homeostasis and contributes to increased inflammatory responses in CF (62). Viruses can also disrupt Ca^2+^ homeostasis by forming membrane structures used for viral particle formation (63). Ivacaftor and lumacaftor normalize mitochondrial and intracellular Ca^2+^ levels and reduce oxidative stress and proinflammatory cytokines levels in CF lungs (64–66). The E protein PBM of SARS-CoV-2 may affect virus replication by altering Ca^2+^ homeostasis, leading to the reduction in SARS-CoV-2 titers observed in cells infected and treated with lumacaftor or ivacaftor (Fig. 8) (67–69). In fact, genes responsible for Ca^2+^ homeostasis, such as ATP2A3 and ATP2B2, also are underregulated due to the presence of E protein PBM from SARS-CoV-2 (Fig. 6C). Complementary studies have demonstrated that the use of Ca^2+^ channel blockers may reduce the mortality from SARS-CoV-2 in humans (70, 71).

In summary, loss of CFTR function in the setting of severe CoV infection may contribute to lung injury through multiple mechanisms including impaired fluid and electrolyte transport, altered Ca^2+^ homeostasis, and NLRP3 inflammasome activation. Ivacaftor and lumacaftor treatment partially reversed this pathology and improved survival in a mouse model of severe disease. FDA-approved CFTR modulators, now widely used for patients with cystic fibrosis, may also help to reduce morbidity and mortality due to high-pathogenicity human coronaviruses.

## MATERIALS AND METHODS

### Ethics statement.

All animal experimental protocols were approved by the NIH-CDC in accordance with US legislation, the Environmental Council of Madrid (permit number: PROEX 146.6/20), and the Ethical Committee of the Center for Animal Health Research (CISA-CSIC) (permit numbers: CBS 2014/005 and CEEA 2014/004), in strict accordance with Spanish National Royal Decree (RD 53/2013) and international European Union guidelines 2010/63/UE on the protection of animals used for experimentation and other scientific purposes and Spanish National law 32/2007 about animal welfare. All work with infected animals was performed in a biosafety level 3+ (BSL3+) laboratory of the Center for Animal Health Research (CISA-CSIC, Madrid, Spain). Infected mice were housed in a self-contained ventilated rack (Allentown, NJ).

### Viruses.

Mouse-adapted SARS-CoV (MA15) (72) parental wild-type (wt) and recombinant viruses were rescued from infectious cDNA clones generated in a bacterial artificial chromosome (BAC). rSARS-CoV-PBM^−^ was generated in our lab previously (15).

### Generation of recombinant virus infectious clones.

The cDNA of SARS-CoV-MA15 assembled in a pBAC (pBAC-SARS-CoV-MA15) (73) was used to introduce the corresponding mutations affecting the E protein of SARS-CoV. To generate viruses with mutations in the SARS-CoV E protein carboxy terminus, DNA fragments including nucleotides 26017 to 26884 of the SARS-CoV-MA15 genome flanked by BamHI and XcmI restriction sites were generated using the oligonucleotides indicated in Table 1. These fragments carried different mutations located on the E gene generating amino acid changes at the E protein carboxy terminus: PBM-MERS, PBM-HKU1, and PBM-OC43. The fragments were digested with enzymes BamHI and XcmI and introduced into an intermediate plasmid pBAC-BamHI*-RsrII*-SARS-CoV containing nucleotides 26044 to 29783 of the SARS-CoV infectious clone. Next, these plasmids were digested with enzymes BamHI and *RsrII*, and the fragments containing the different mutations were inserted into plasmid pBAC-SARS-CoV-MA15 digested with the same enzymes, obtaining the respective infectious clones. The integrity of cDNA was verified by restriction analysis with the enzyme HindIII and by Sanger sequencing.

**TABLE 1:** PCR primers used to engineer SARS-CoV-E-PBM mutants

Virus/primer	Sequence (5′→3′)
PBM-MERS	
SARS-E-VS	CTCTTCAGGAGTTGCTAATCCAGCAATGG
SARS-E-PBM-MERS-RS	TTAGAACCATTCATCAGGAACTCCTTCAGAAGAGTTCAG
SARS-E-PBM-MERS-VS	TCTGAAGGAGTTCCTGATGAATGGTTCTAAACGAACTAACTATTA
SARS-26885-RS	GGTCCTTAATGTCACAGCGCCC
PBM-HKU1	
SARS-E-VS	CTCTTCAGGAGTTGCTAATCCAGCAATGG
SARS-E-PBM-HKU1-RS	CGTTTAGATCAGATCATCAGGAACTCCTTCAGAAGAGTT
SARS-E-PBM-HKU1-VS	GAAGGAGTTCCTGATGATCTGATCTAAACGAACTAACTATTA
SARS-26885-RS	GGTCCTTAATGTCACAGCGCCC
PBM-OC43	
SARS-E-VS	CTCTTCAGGAGTTGCTAATCCAGCAATGG
SARS-E-PBM-OC43-RS	TTAGACGTCATCAACAGGAACTCCTTCAGAAGAG
SARS-E-PBM-OC43-VS	TCTGAAGGAGTTCCTGTTGATGACGTCTAAACGAACTAACTATTA
SARS-26885-RS	GGTCCTTAATGTCACAGCGCCC
MERS-CoV PBM−	
SA27502VS	GCTTATCGTTTAAGCAGCTC
Rs mutPBM	CATATTAGACATTATGAAGGAGTTCGTTATCCTCCTCCTCCAGGTGGT
VS mutPBM	AACCCCCTCTACCACCTGGAGGAGGAGGATAACGAACTCCTTCATAAT
SA28319RS	TCTGTCGTAGTCACAAGCAC

SARS-CoV-2 cDNA was assembled in a pBAC (pBAC-SARS-CoV-2) following previously described procedures (13, 74, 75). This infectious cDNA clone was used to introduce the corresponding mutations affecting the PBM of the SARS-CoV-2 E protein. First, a DNA fragment was obtained by chemical synthesis (GeneScript) that included nucleotides 26386 to 26751 of the SARS-CoV-2 genome, flanked by HpaI and AgeI restriction sites. This fragment carried the carboxy terminus mutation eliminating the PBM (DLLV → GGGG). The fragment digested with HpaI and AgeI was introduced into the corresponding sites of plasmid psL-F6-SARS-CoV-2, which contains nucleotides 25314 to 28610 of the SARS-CoV-2 genome, generating plasmid psL-F6-SARS-CoV-2-E-PBM^−^. Plasmid psL-F6-SARS-CoV-2-E-PBM^−^ was then digested with restriction enzymes BamHI and AvrII and the resulting fragment was cloned into an intermediate plasmid pBAC-F6-SARS-CoV-2. Subsequently, this plasmid with was digested with BamHI and *RsrII* and the fragment containing the E gene mutation was inserted into the unique BamHI and *RsrII* cloning sites of the pBAC-SARS-CoV-2 plasmid to generate the corresponding cDNA infectious clone. The integrity of the cloned DNA was verified by restriction analysis with the enzyme EcoRI and by Sanger sequencing.

The cDNA infectious clone of MERS-CoV strain EMC/2012, assembled in pBAC (pBAC-SA-FL) (13), was used to generate the mutants with a modified MERS-CoV E protein PBM. First, DNA fragments including nucleotides 27502 to 28319 of the MERS-CoV genome flanked by *KflI* and *Pfl23II* restriction sites were generated by PCR using the oligonucleotides indicated in Table 1. The fragment obtained was introduced into an intermediate plasmid pBAC-SA-F6 including nucleotides 25841 to 30162 of the MERS-CoV genome using the restriction enzymes *KflI* and *Pfl23II*. The 25,841 to 30,162 region of the MERS-CoV genome is flanked by the PacI and RsrII unique restriction sites in plasmids pBAC-SA-FL and pBAC-SA-F6. The PacI*-RsrII* region of intermediate plasmid pBAC-SA-F6, with the desired E gene mutation, was cloned into pBAC-SA-FL to generate the complete infectious cDNA clone of MERS-CoV including E protein PBM mutations. All cloning steps were verified by Sanger sequencing.

### Recovery of recombinant virus variants from cDNA clones.

Vero E6, Huh-7, and Vero E6-TMPRSS2 cells were grown to 95% confluence in 12.5 cm^2^ flasks and transfected with 6 μg of each infectious SARS-CoV, MERS-CoV, and SARS-CoV cDNA clones, and 18 μL of Lipofectamine 2000 (Invitrogen), according to the manufacturer’s specifications. At 6 h posttransfection the transfection medium was replaced by standard Dulbecco’s medium with 10% fetal calf serum and incubated at 37°C for 72 h. Cell supernatants were harvested and passaged once on fresh cells, and the recovered viruses were cloned by three rounds of plaque purification, following standard procedures for SARS-CoV. In the case of MERS-CoV and SARS-CoV-2, the supernatants were collected at 72 h posttransfection and titrated by plaque lysis formation in semisolid medium. Viruses were then amplified in 75 cm^2^ flasks, by infecting monolayers of the cells indicated for each virus at a multiplicity of infection (MOI) of 0.001. The supernatants were collected at 72 hpi and titrated. The 3′-end genome of different viruses, from the end of the replicase to the untranslated region, was sequenced. All sequences were compared to that of the parental wt virus sequence using SeqMan software (Lasergene, Madison, WI).

### Growth kinetics.

Subconfluent monolayers (around 90% confluence) of Vero E6 and Huh-7 cells were infected at an MOI of 0.001 with parental viruses or the respective mutant viruses without E protein PBM. Culture supernatants were collected at 24, 48, and 72 hpi, and virus titers were determined as previously described (12).

### Mice.

To determine mutant virus pathogenesis, SARS-CoV, MERS-CoV, and SARS-CoV-2 viruses were inoculated into the corresponding mice strains: 16-week-old BALB/c OlaHsd (Harlan), K18-hDPP4 (76), and K18-hACE2 (77) (Jackson Laboratories), respectively. Mice were anesthetized with isoflurane and inoculated intranasally using 100,000 or 10,000 PFU of the corresponding virus in a 50 μL volume of DMEM supplemented with 2% FBS. Manipulation of infected mice was carried out in a level 3+ biological containment laboratory at CISA-CSIC (Algete, Madrid) equipped with the required containment infrastructure for animal and cell cultures work. Scores for clinical signs of disease were determined based on the animal physical appearance and behavior. They were classified in five grades of severity (0, normal strength; 1, less active; 2, dull or sluggish movements, ruffled fur, squinting eyes; 3, ataxia, tremors; and 4, motionless, lying down, or hunched posture). Virus titrations were performed as previously described (12). Viral titers were expressed as PFU counts per gram of tissue.

### Lung viral titers of infected mice.

Lungs from infected mice were harvested on different days postinfection. Lungs were homogenized in 2 mL of PBS containing 100 IU/mL penicillin, 0.1 mg/mL streptomycin, 50 μg/mL gentamicin, and 0.5 μg/mL amphotericin B (Fungizone), using gentleMACS Dissociator (Miltenyibiotec). Virus titers were determined as previously described (12).

### Lung histopathology.

The entire left lung lobe was removed from each mouse and immersion-fixed in zinc formalin (Sigma-Aldrich) for 48 h. After the fixation period, samples were routinely processed and embedded in paraffin blocks that were then sectioned at a 4-μm thickness on a microtome, mounted onto glass slides, and routinely stained with hematoxylin and eosin (H&E). Lung sections were microscopically evaluated using an Olympus BX43 microscope by a single veterinary pathologist who was blinded to the identity of mice (Veterinary Pathology Department, Animal Health Research Center-CISA-CSIC, Valdeolmos, Spain). To assess the presence and severe histopathological lesions, lung inflammation parameters based on previous reports on SARS-CoV-2 infection in mouse models were used (78). The histopathological parameters evaluated were as follows: alveolar hemorrhage, alveolar edema, perivascular edema, alveolar septal thickening (interstitial pneumonia), inflammatory cell infiltration in alveoli, bronchi/bronchioles with epithelial necrosis, detached epithelium or inflammatory cells in the lumen (bronchitis/bronchiolitis), peribronchial/peribronchiolar and perivascular mononuclear infiltrates, and cytopathic effect in pneumocytes or syncytium formation. The histopathological parameters were graded following a semiquantitative scoring system as follows: 0, no lesion;1, minimal lesion; 2, mild lesion; 3, moderate lesion; and 4, severe lesion. The cumulative scores of histopathological lesions provided the total score per animal. In each experimental group, the individual scores were used to calculate the group average.

### Extraction and analysis of RNA.

Cell culture or lung RNA samples were collected and incubated in RNAlater reagent (Ambion). To extract total RNA, lungs were homogenized in 2 mL of RLT lysis buffer (Qiagen) supplemented with 1% β-mercaptoethanol using the GentleMACS Dissociator system (Miltenyi Biotec) following the protocol proposed by the manufacturer. RNA was purified from supernatants using the rNeasy minikit reagent (Qiagen), as previously described. To generate cDNAs from the purified RNAs, a reverse transcription (RT) reaction was carried out using the High Capacity cDNA RT kit reagent (Applied Biosystems). To verify the sequence of the mutations introduced into the viral genome, 4 μL of the cDNA generated in the RT reaction was used as a template in a PCR using the enzyme *Taq* polymerase (Invitrogen). Cellular mRNA expression was analyzed by quantitative reverse transcription-quantitative PCR (RT-qPCR), using TaqMan technology with commercial probes (ThermoFisher Scientific) (Table 2). In all cases, the reaction was performed with the FastStart Universal Probe Master reagent (Rox) (Roche). qPCRs were performed in a 7500 Real Time PCR System (ThermoFisher Scientific). qPCR data were analyzed using the 7500 software v2.0.6 (ThermoFisher Scientific). All experiments met the recommendations for analysis of gene expression by RT-qPCR (MIQE) (79). Each result is the average of three independent experiments in which each sample was tested in triplicate. The relative quantification of gene expression was performed from the mean values of *CT* (cycle in which the amplification curve cuts at the threshold level using the 2^−ΔΔ^*^CT^* method) (80). The level of 18S rRNA was used as an endogenous control to normalize cellular mRNA levels in mouse lungs, while hydroxymethylbilane synthase (HMBS) was used to normalize cellular mRNA levels in cell cultures.

**TABLE 2:** TaqMan probes used to quantify cellular mRNAs by RT-qPCR

Gene	TaqMan assay ID
mCFTR	Mm00445197_m1
hCFTR	Hs00357011_m1
mNLRP3	Mm00840904_m1
mTNF-α	Mm00443258_m1
mIL-6	Mm00446190_m1
mIL-1β	Mm01336189_m1
mCXCL10	Mm00445235_m1
mCXCL2	Mm00436450_m1
18S rRNA	Mm03928990_g1
HMBS	Hs00609297_m1

### Deep sequencing of mRNAs.

Total RNA extracted from the lungs of infected mice was used for rNaseq sequencing. The samples were digested with the DNAsaI (Roche) to eliminate possible DNA contamination. RNA was processed to remove rRNA, a major component in large RNA samples, and enrich the resulting sequences for less abundant expressed RNAs such as mRNAs. RNA libraries were constructed (Illumina method) and 300 nt paired-end reads were obtained in which the complementary strand was marked to distinguish the original sense of the sequences. Three replicates per variable and per day were sequenced. From each sample, 80 million sequences were obtained.

### Genomic alignment of Illumina paired-end reads.

Paired-end reads in FASTQ format were quality checked with FASTQC (www.bioinformatics.babraham.ac.uk/projects/fastqc; 2021). As no limitations were detected for each sample, all unprocessed reads were aligned to the mouse genome (GRCm38, primary assembly) using RNA-STAR (81) (–alignIntronMax 1000000 –alignMatesGapMax 1000000). Duplicated reads (i.e., those aligned at identical genomic positions) were marked using MarkDuplicates function of picard-tools (https://broadinstitute.github.io/picard/; 2021). Alignment files in SAM format were compressed, sorted, and indexed with corresponding commands of Samtools package (82). The IGV browser (83) was used to visualize alignment results.

### Quantification of mouse genes and differential expression determination.

All mouse genes (ENSEMBL release 101) were quantified using featureCounts function of bioconductor package Rsubread (84) (ignoreDup=TRUE, requiredBothEndsMapped=TRUE, primaryOnly=TRUE). To calculate differential expression between pairs of replicated samples, DESeq and lfcShrink functions of bioconductor package DESeq2 (85) were used (cooksCutoff=FALSE, independentFiltering=TRUE, type=”normal”). Differential expression results, including adjusted *P* values by FDR (86), were stored as text-tabulated files and converted to FIESTA format (https://bioinfogp.cnb.csic.es/tools/FIESTA; 2021) to facilitate the visualization of overexpressed and underexpressed genes in form of MA plots. For each comparison, genes with |logRatio| ≥ 1.5 and FDR ≤ 0.05 were considered differentially expressed.

### Functional analysis of relevant genes.

The DAVID tool (87) was used to determine overrepresentation of Gene Ontology terms (Biological Process) in differentially expressed genes (both overexpressed and underexpressed). The six most representative biological processes were selected based on the *P* value.

### Treatment of mice with CFTR modulators.

Mice (*n* = 11) were treated by intraperitoneal injection administration with an appropriate concentration of compounds 24 h before infection with 10,000 PFU of SARS-CoV-2. Mock-infected or infected animals were treated once daily with 12 or 6 mg/kg of Lumacaftor or Ivacaftor, respectively. All treatments ceased after day 8 postinfection. Three mice from each group were euthanized at 3 and 6 dpi to analyze virus production in the lungs.

### Cell viability assay.

To determine the cytotoxicity of commercial compounds that modulate CFTR protein activity, cells were incubated with different concentrations (1 to 50 μM) of ivacaftor (VX-770, Merck) or lumacaftor (VX-809; Selleck Chemicals) and cell viability was determined by the MTT [3-(4,5-dimethylthiazol-2-yl)-2,5-diphenyltetrazol bromide] reduction assay (88).

### Statistical analysis.

Two-tailed, unpaired Student’s *t* tests were used to analyze the differences in mean values between groups. The statistical significances are indicated in the figures as follows: *, *P < *0.05; **, *P < *0.01; ***, *P < *0.001.
