# Supplementary figures and images for: SARS-CoV-2-Mediated Lung Edema and Replication Are Diminished by Cystic Fibrosis Transmembrane Conductance Regulator Modulators
Source: mBio. 2023 Jan 10;14(1):e03136-22. doi: 10.1128/mbio.03136-22 (PMC9973274; doi:10.1128/mbio.03136-22)

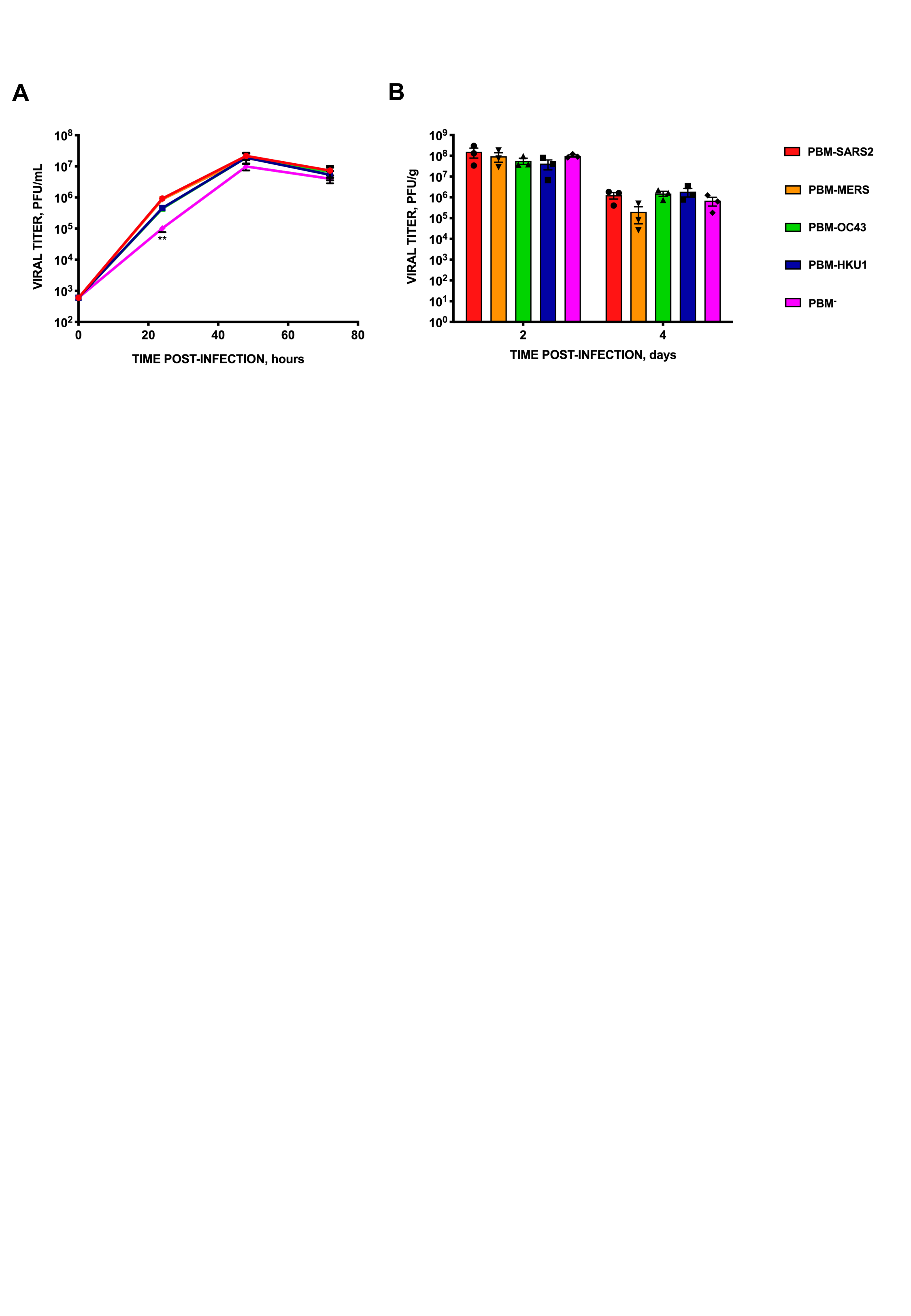

Supplement: FIG S1 [file mbio.03136-22-s0001.tif]

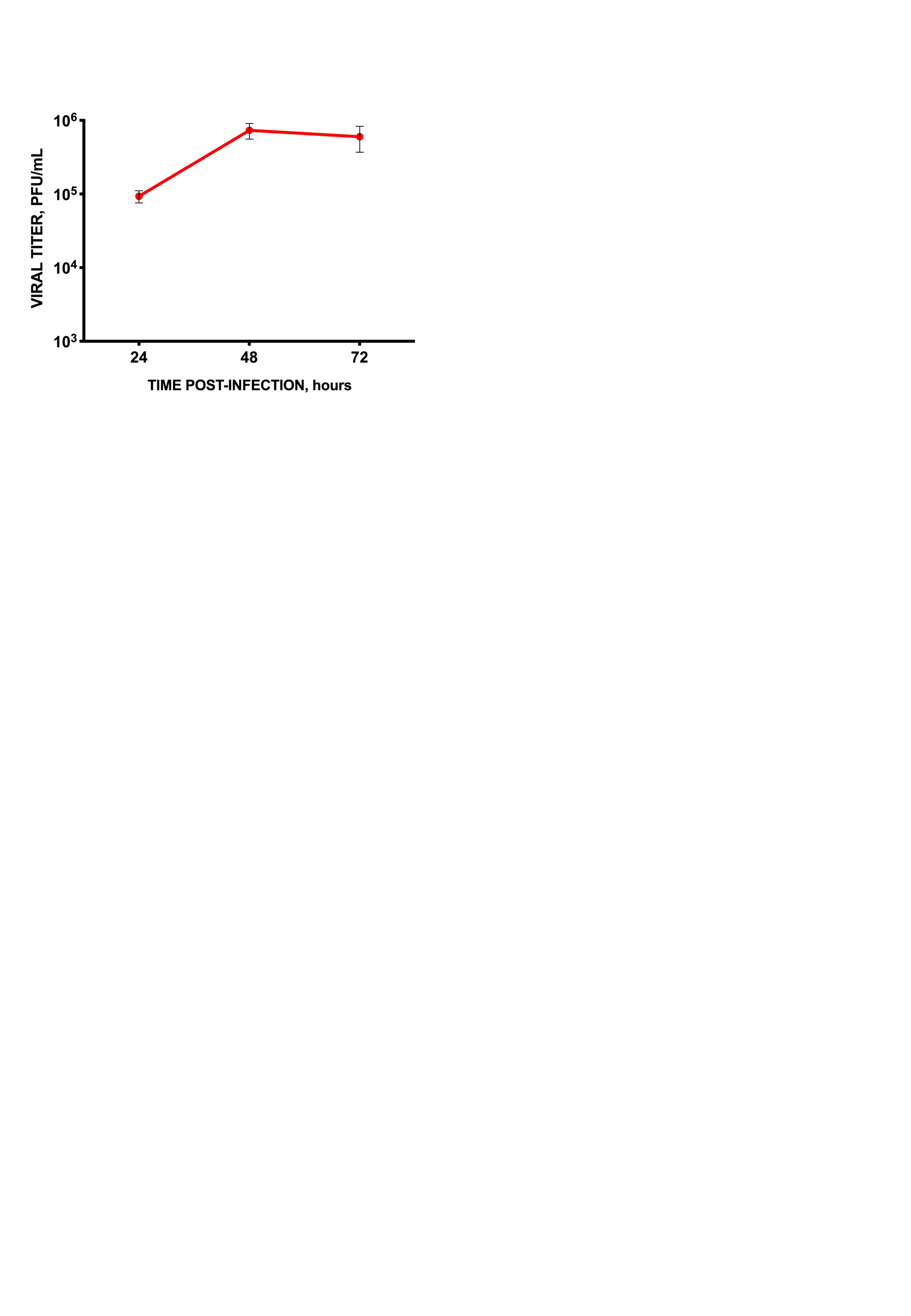

Supplement: FIG S2 [file mbio.03136-22-s0002.tif]

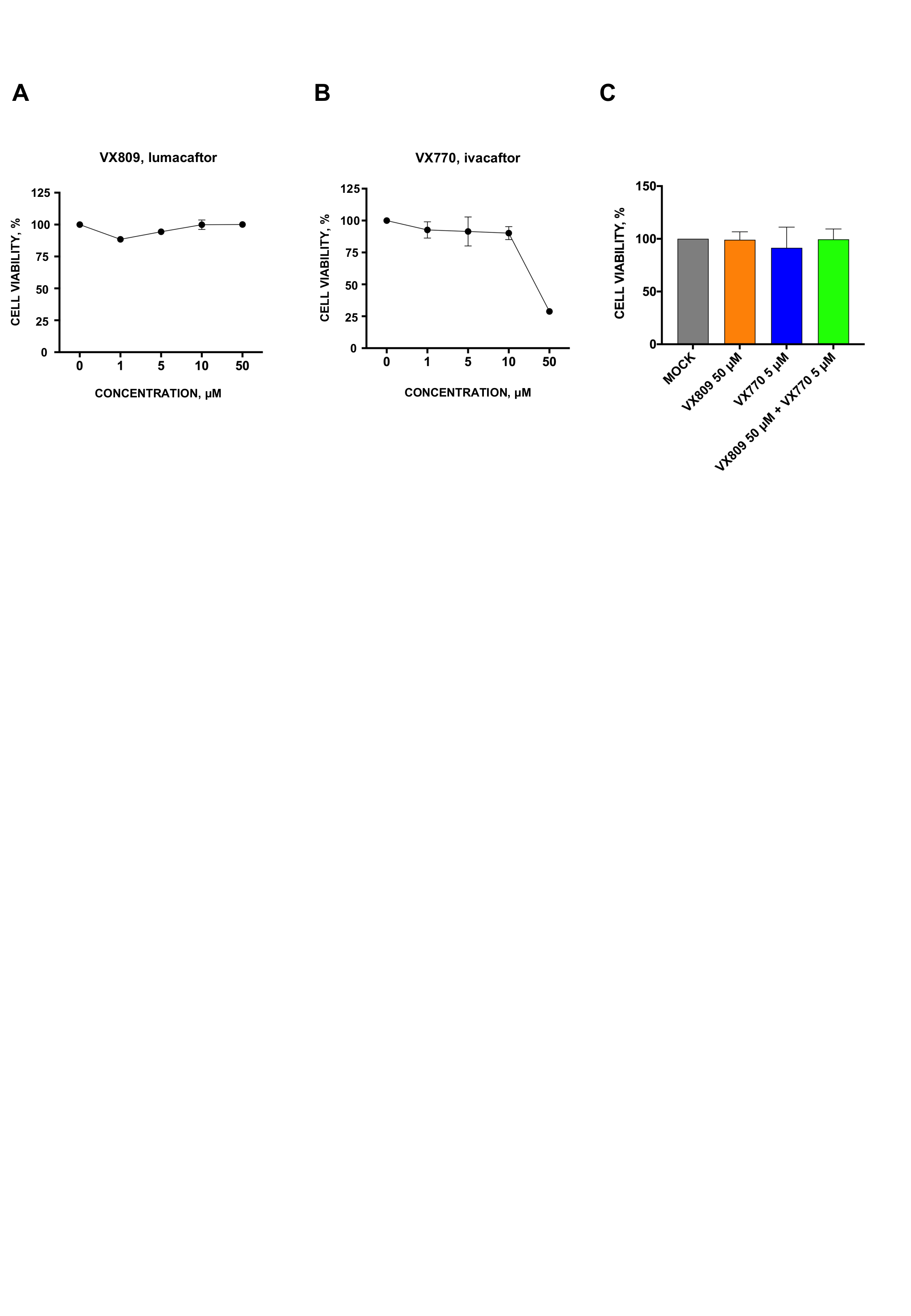

Supplement: FIG S3 [file mbio.03136-22-s0003.tif]
